# Truth telling and communication skills

**DOI:** 10.4103/0019-5545.55098

**Published:** 2009

**Authors:** S. K. Chaturvedi

**Affiliations:** National Institute of Mental Health and Neurosciences, Bangalore - 560 029, India. E-mail: skchatur@gmail.com

Sir,

This interesting topic initiated by Swaminath[1] and taken forward by Shamsundar[2] is of great clinical significance especially when dealing with persons suffering from terminal or incurable diseases. A lot of research has been done on communication skills with respect to breaking bad news and dealing with collusion within the family. Shamsundar[2] wonders if there are any well laid-out, generally agreed upon guidelines on how to go about this task of ‘truth telling’. Currently, some guidelines based on research and experiences have been described.

SPIKES, a six-step method[3] of breaking bad news or truth telling, suggests the six *s*teps as setting up the interview, assessing the patient's *p*erception, obtaining the patient's *i*nvitation, giving *k*nowledge and information to the patient, addressing patient's *e*motions and empathic responses, and *s*trategy and summarizing. Other guidelines have been provided by Peter Kaye,[4] Brewin,[5] Maguire[6] and Faulkner and Maguire.[7] Similar guidelines for oncology and palliative settings, for India, have also been described.[8,9] The McMaster technique[8] is another step wise method which can be adapted with our socio cultural background.

Using the above strategies, the situation in Narayan's[10] “The doctor's word”, described by Swaminath,[1] could have been handled differently to avoid the conspiracy of silence, deception, collusion, and the ‘cost’ of deception and collusion. The doctor chooses ‘not to tell’ his patient-friend in order to protect him from distress or being shattered. The doctor could have checked, if his patient-friend already knew or had some idea about his critical condition, and informed him gradually after a warning shot, in a step by step way and, more importantly, handled the emotions or distress which followed, maintaining hope. Any urgent unfinished business, like writing the will, could have been completed. The doctor's patient-friend would have felt reassured with the support, maintained trust in his doctor friend and still survived. By providing hope through deception, the doctor loses his patient-friend's trust in him, and maybe even loses the friendship of his patient- friend, who survived. The doctor also puts himself at risk of potential guilt in case the patient-friend did not survive. Further, the absence of a ‘written will’ would give survivors enormous legal difficulties.

The tasks of truth telling are no doubt challenging and the physician should acknowledge that it leaves an impact on self as well. The advantage of telling the truth is that one does not need to remember what one has told, whereas one has to clearly remember the exact deceptive information given, in order to avoid being caught, and have a loss of trust and loss of face!

## References

[1] Swaminath G (2008). The doctor's dilemma: Truth telling. Indian J Psychiatry.

[2] Shamasundar C (2008). Telling the truth to patients and relatives. Indian J Psychiatry.

[3] Baile WF, Buckman R, Lenzi R, Glober G, Beale EA, Kudelka AP (2000). SPIKES-A six-step protocol for delivering bad news: Application to the patient with cancer. Oncologist.

[4] Kaye P (1995). Breaking bad news.

[5] Brewin T (1991). Three ways of giving bad news. Lancet.

[6] Maguire P (1998). Communicating with cancer patients.

[7] Faulkner A, Maguire P (1994). Talking to cancer patients and their relatives.

[8] Chaturvedi SK, Chandra PS, Simha S (2008). Communication skills in palliative care.

[9] Chaturvedi SK, Chandra P, Chandra PS, Chaturvedi SK (1998). Dealing with difficult situations. Psycho Oncology: Current Issues.

[10] Narayan RK (2006). The doctor's word. In Malgudi Days. Penguin Twentieth Century Classics. Paperback.

